# Revealing the Crystal Structure of the Purine Base Xanthine with Three-Dimensional (3D) Electron Diffraction

**DOI:** 10.1021/acs.cgd.4c01594

**Published:** 2025-02-11

**Authors:** Helen W. Leung, Royston C. B. Copley, Giulio I. Lampronti, Sarah J. Day, Lucy K. Saunders, Duncan N. Johnstone, Paul A. Midgley

**Affiliations:** † Department of Materials Science and Metallurgy, University of Cambridge, 27 Charles Babbage Road, Cambridge, CB3 0FS, United Kingdom; ‡ GSK R&D, Gunnels Wood Road, Stevenage, SG1 2NY, United Kingdom; § Diamond Light Source, Ltd., Beamline I11, Harwell, Oxford, OX11 0DE, United Kingdom

## Abstract

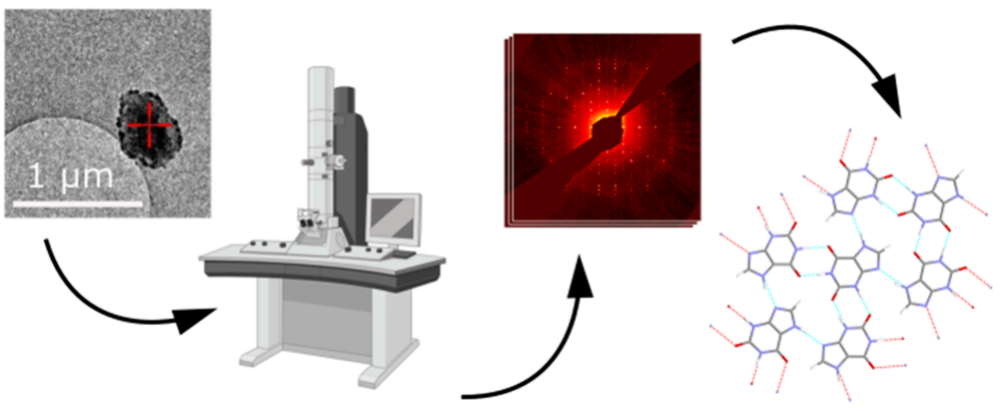

Three-dimensional (3D) electron diffraction (3D-ED) techniques can be used for structure determination, circumventing challenges posed to conventional and bulk X-ray diffraction techniques such as submicrometer-sized crystals, the strong effects of texture, the presence of defects, and polyphasic samples. Such challenges previously prevented the structure solution of xanthine, a purine base chemically similar to guanine that may also be found in organisms. In this work, we use 3D-ED to elucidate the crystal structure of xanthine. The electron diffraction data obtained from a single microcrystal is also of sufficient quality to determine hydrogen positions, confirming the presence of the 7*H*-tautomer, as expected. This study highlights the potential for the use of 3D-ED on biogenic nanocrystals, for example opening opportunities to understand the links between crystal anisotropy, birefringence, and organism characteristics.

## Abstract 2

3D electron diffraction (3D-ED) is used for structure determination, circumventing challenges posed to conventional X-ray diffraction techniques. We use 3D-ED to elucidate the crystal structure of xanthine, a purine base. The data are of sufficient quality to determine hydrogen positions, confirming the presence of the 7*H*-tautomer. This study highlights the potential for the use of 3D-ED on biogenic nanocrystals.

In recent years, three-dimensional electron diffraction (3D-ED) techniques have emerged as a powerful tool for use in structure determination. While conceptually analogous to single-crystal X-ray diffraction (SCXRD), 3D-ED can overcome many of its limitations due to the strong interaction of electrons with the crystal potential. Combined with the use of focused electron probes, microcrystals up to a million times smaller in volume than would be needed for SCXRD can be studied.^1^ This addresses challenges in materials that are scarce, or where larger single crystals cannot be obtained. 3D-ED has become increasingly well-established for determining the structure of organic crystals in the past decade.^2,3^

Biogenic crystals are widely produced by animals for a range of functions such as forming iridescent structures, e.g., scales, feathers, shells,^4^ and reflective structures in eyes to improve vision in low-light environments by reflecting light back into the retina.^5^ The majority of biogenic crystals are made of guanine, a nucleotide base in deoxyribonucleic acid (DNA). The origin of the optical properties of these biogenic guanine crystals can be explained by understanding their crystal structure, which consists of densely stacked hydrogen-bonded layers.^6,7^ The platelike morphology of guanine crystals formed in organisms leads to a high refractive index face with significant reflectivity for wavelengths in the range of visible light. Periodic variations in the refractive index (resulting from birefringence) leads to constructive interference, high reflectivity,^8^ and the ability to scatter light to generate structural colors.^9^

Xanthine (3,7-dihydropurine-2,6-dione) is a member of the purine family and consists of fused five- and six-membered, heterocyclic rings (Figure 1a). This chemical “skeleton” is shared with other purines such as hypoxanthine^10^ (Figure 1b), guanine^11^ (Figure 1c), and adenine^12^ (Figure 1d). Xanthine itself is a metabolic intermediary produced in purine degradation and a precursor in the synthesis of uric acid. It can therefore be found widely in organisms and their waste products. Recently, xanthine has also been found in the form of preferentially oriented layers of biocrystals appearing to back-reflect light into receptors in the median ocellus in the *Archaeognatha* insect group.^13,14^ Given that the majority of biogenic crystals are made of guanine, it is currently unclear why xanthine may sometimes be found instead.^14^ Understanding the structure of xanthine crystals will give insight into its role and optical properties and how this might differ from the guanine crystal function, providing a greater understanding of why xanthine may form preferentially over guanine.

**Figure 1:**
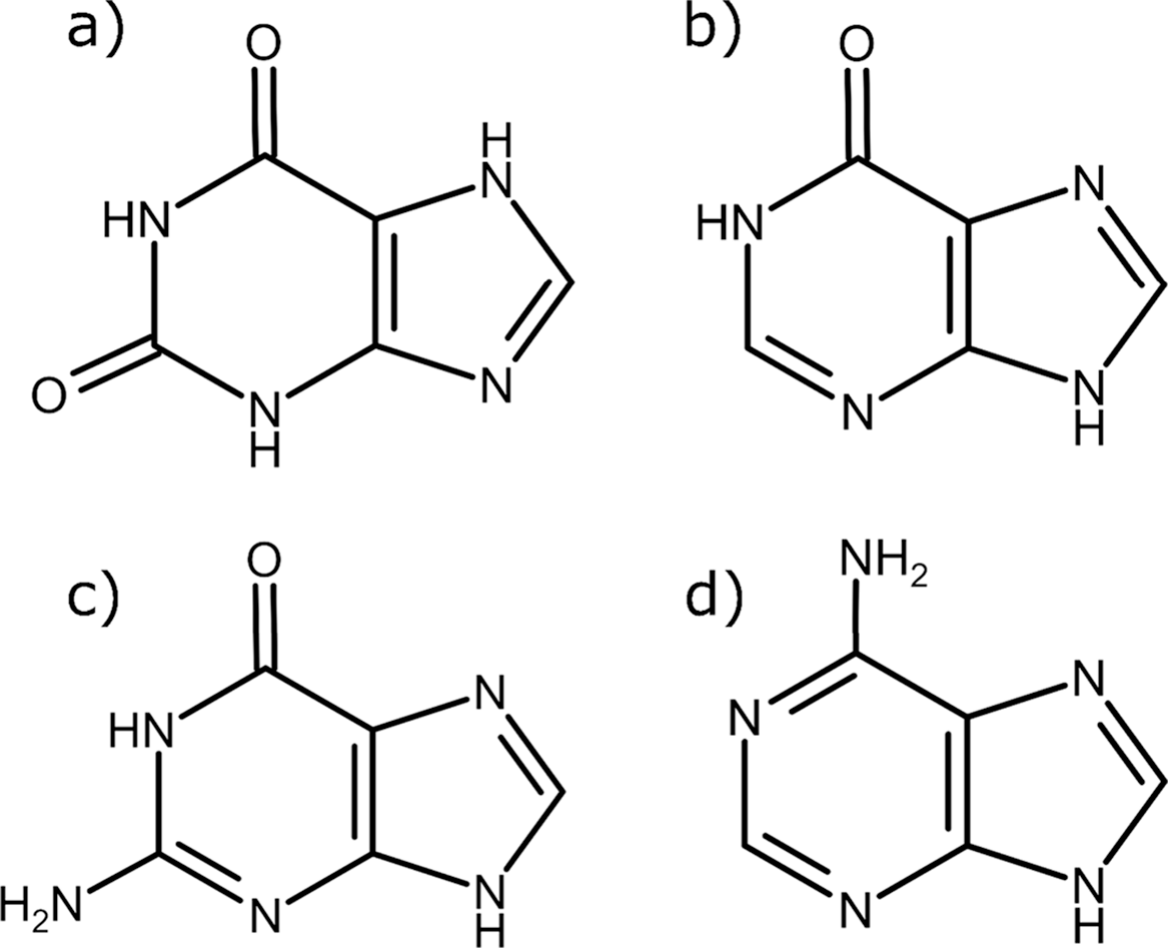
(a) The xanthine molecule (C_5_H_4_N_4_O_2_). The 7*H*-tautomer is shown (the numbering scheme is shown in Figure S3.1): this is the prevalent tautomer and the expected chemical structure from deamination of guanine (which is how the synthetic xanthine sample, purchased from Sigma–Aldrich for this work, was produced^22^). (b) The hypoxanthine molecule (C_5_H_4_N_4_O). (c) The guanine molecule (C_5_H_5_N_5_O). Due to their chemical similarities, it has been previously postulated that xanthine should have a crystal structure similar to that of guanine.^11^ (d) The adenine molecule (C_5_H_5_N_5_).

Xanthine is also a precursor in the synthesis of a range of biorelevant molecules.^15^ Its derivatives include theophylline (widely used in the treatment of respiratory conditions^16^), caffeine (a well-known central nervous system stimulant^17^), and theobromine (the primary alkaloid found in cocoa^18^). As such, xanthine’s use as a scaffold in the synthesis of its biologically active derivatives has also been highlighted to be of interest.^19^ Aside from biorelevant applications, the role of synthetic xanthine as an effective nucleator in the epitaxial nucleation of polyhydroxyalkanoate (PHA) polymers has also been investigated. PHA polymers are of interest because they are known to degrade in every natural environment on Earth: knowledge of the crystal structure of xanthine could provide further insight into its role as a naturally occurring nucleating agent for this purpose.^20^

Despite being such a fundamental molecule, the crystal structure of xanthine remains unknown, with crystals too small to be used for SCXRD and complexities arising from strong preferred orientation of crystals affecting powder XRD studies. Recently, the potential for 3D-ED to be applied to purine base compounds has been demonstrated with its use to confirm the SCXRD structure of β-guanine from biogenic samples.^21^ In addition, electron diffraction experiments using conventional transmission electron microscopy (TEM) techniques to probe xanthine crystals have predicted a structure similar to that of guanine crystals.^11,14^

In this work, we use 3D-ED to solve the previously unknown crystal structure of synthetic xanthine from a single submicrometer-sized particle found in a commercial powder sample. This result demonstrates the potential of the technique for structure elucidation of scarce biogenic xanthine samples to advance exploration in the field of organic biomineralization. In addition, we show that the quality of the electron diffraction data is sufficient to refine hydrogen atom positions, using kinematical 3D-ED techniques.

## Results and Discussion

**Figure 2:**
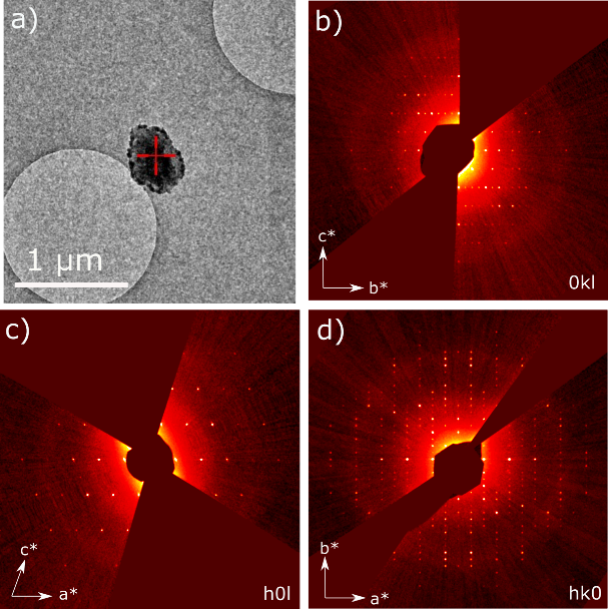
(a) Microcrystal of xanthine from which structure solution was successful. Some amorphous contamination is overlaid on top of this crystal while the crystal itself can be seen as a darker outline with straight edges, ∼300 nm wide. The amorphous carbon coating on the grids and the contamination both contribute to inelastic scattering which leads to a diffuse halo seen across each diffraction pattern. (b) 0*kl*, (c) *h*0*l*, and (d) *hk*0 slices from reconstructed reciprocal space. Systematic absences are consistent with the *P*2_1_/*c* space group. Figure S2.1 shows the slices with an overlaid reciprocal lattice grid.

Xanthine powder was purchased from Sigma–Aldrich and was deposited onto Quantifoil R1.2/1.3 grids, as described in Note S1 in the Supporting Information. TEM images of xanthine particles revealed a typically block-like morphology (Figure S1.1). 3D-ED data were collected from microcrystals using a Thermo Fisher Titan Krios G3i transmission electron microscopy (TEM) system operated at 300 kV and with a CETA-16 M camera (further details are given in Notes S2 in the Supporting Information). The tilt series was used to reconstruct the 3D reciprocal space lattice, giving a monoclinic unit cell [*a* = 9.82(11) Å, *b* = 17.87(8) Å, *c* = 6.79(13) Å, β = 107.5(9)°, *V* = 1136(26) Å^3^]. Systematic absences from reciprocal space sections (Figures 2b–d) were used to deduce the likely space group of the structure and are consistent with *P*2_1_/*c* (unique *b*-axis). Upon integration, a dataset from a single crystal ∼300 nm wide (Figure 2a) led to a completeness of 82.0% to a resolution of 0.8 Å. This was sufficient to allow a successful structure solution without merging datasets from different crystals.

**Figure 3:**
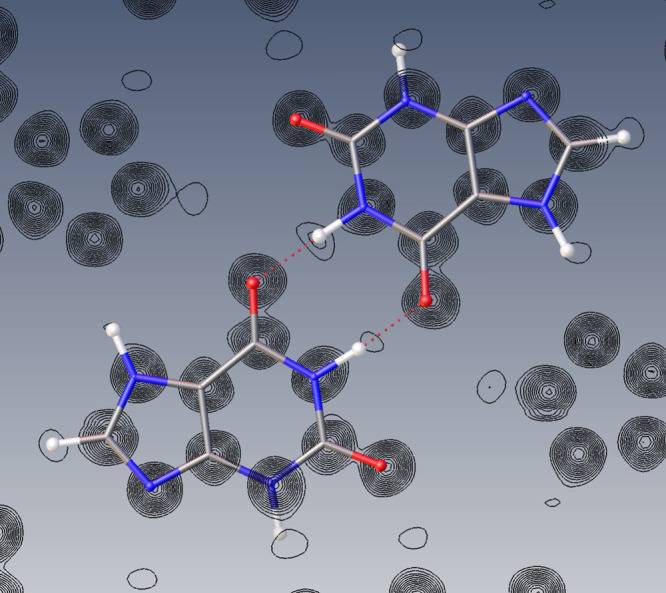
A Fourier electrostatic potential (*F*_obs_) map generated through the mean plane of the independent molecules (∼(101̅)) supports the successful identification of hydrogen atom positions within the xanthine molecule, confirming the presence of the 7*H*-tautomer. *F*_obs_ refers to the observed structure factors. Contours represent the calculated electrostatic potential for a mean plane taken through both independent molecules. There is some deviation of the molecules from the mean plane; hence some atoms are displayed “below” the contours. Only positive electrostatic potential is displayed. Steps between contour lines represent a 7% step in electrostatic potential. Atoms are colored as follows: nitrogen (blue), oxygen (red), carbon (gray), and hydrogen (white). Generated using Olex2.^28^

Structure solution was successful using the ab initio dual space methods implemented in SHELXD.^23^ Initial solutions in the *P*2_1_/*c* space group (*Z* = 8, *Z*′ = 2) provided a structure in which all nonhydrogen atoms were found, giving the correct molecular connectivity for two crystallographically independent xanthine molecules. The structure was then kinematically refined using full-matrix least-squares in SHELXL.^24^ A difference Fourier synthesis (shown in Figure 3) revealed the positions of all of the hydrogen atoms, avoiding the need to place these in geometrically idealized positions (as is often the case for 3D-ED structures).

The coordinates and an isotropic atomic displacement parameter were refined for all non-hydrogen atoms. Ellipsoids showing isotropic refinement of atomic displacement parameters are shown in Figure S3.2. Intensity statistics (specifically, *R*_sigma_^1^ values for different resolution bins) were used to assess the quality of the data and therefore an appropriate cutoff resolution for the final refinement. Based on this, the refinement resolution was truncated to 0.90 Å (corresponding to *R*_sigma_ = 0.33), resulting in an *R*-factor of 10.90%. Table S3.1 contains all relevant crystallographic information.

The successful identification of hydrogen positions unambiguously confirmed the presence of the expected 7*H*-tautomer (the numbering scheme is shown in Figure S3.1). This tautomer is formed upon deamination of guanine and generally predominates over the 9*H*-form of xanthine.^22^Figure 3 shows a Fourier electrostatic potential map in which the hydrogen positions can be clearly recognized. The unrestrained N–H and C–H bond lengths were found to vary between 1.05(3) and 1.10(2) Å (Table S3.2). We note that these bond lengths are longer than the corresponding distances found using X-ray crystallography, particularly since the latter are often used for kinematically refined 3D-ED structures where hydrogen positions cannot be resolved. Locating the position of hydrogen atoms from X-ray data can be complicated by their low atomic number, low scattering power, and variability due to thermal motion (characterized by atomic displacement factors).^25^ In addition, by virtue of having only one electron, a hydrogen atom’s entire electron density is always involved with bonding, resulting in a measured shift of the hydrogen atom toward the atom to which it is bonded using X-ray diffraction data.^26^ It has previously been possible to determine and dynamically refine hydrogen positions with electron diffraction data in which average C–H bond lengths were found to be 1.15 Å by Palatinus et al.^27^ The elongated N–H and C–H distances (in comparison to X-ray distances) discovered here are consistent with trends in this previous work. This observed difference in distances between electron and X-ray diffraction data is likely due to the fundamentally different nature of their interactions, where electrons interact with the crystal potential while X-rays interact with the electron density.

The structure solution and refinement executed here makes use of workflows which come from X-ray crystallography protocols in which electron scattering factors are initially provided (*International Tables*, Vol. C: Tables 4.2.6.8 and 6.1.1.4). Subsequent Fourier synthesis is performed from calculated electron structure factors, giving an electrostatic potential map (Figure 3).

**Figure 4:**
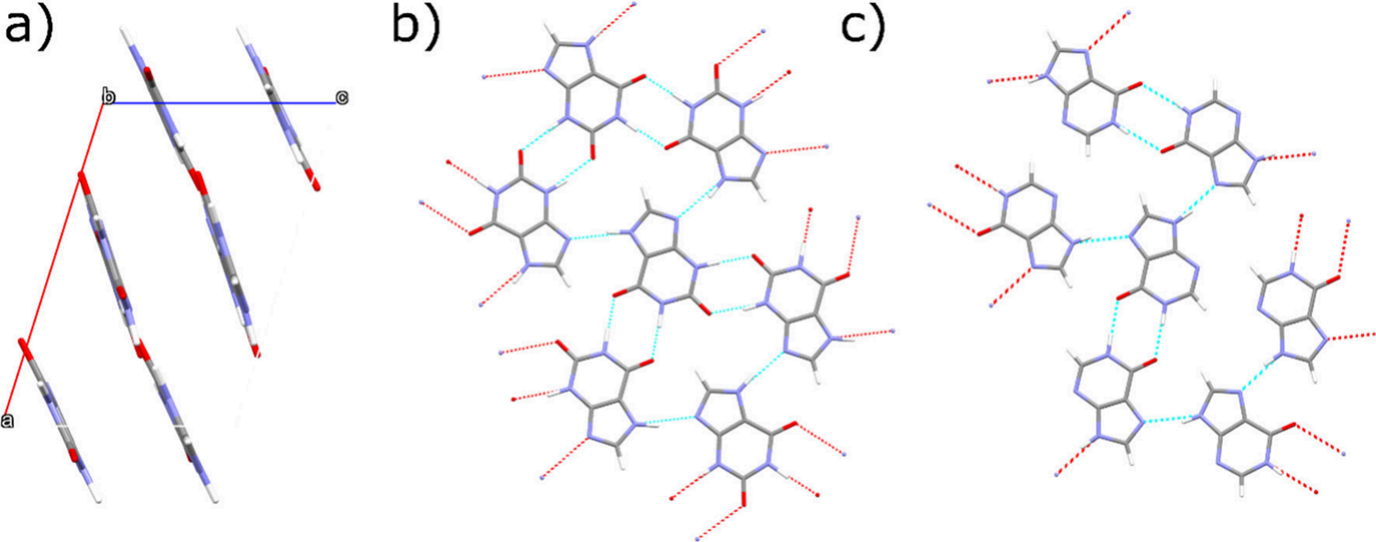
Xanthine structure showing nitrogen (blue), oxygen (red), carbon (gray), and hydrogen (white) atoms. (a) The view looking down the *b*-axis. (b) The hydrogen-bonded layers of xanthine molecules, defined by the (202̅) planes. (c) Hydrogen bonding in hypoxanthine. Structures are visualized using the Mercury software.^29^

The xanthine crystal structure contains a two-dimensional hydrogen bonded network (Figure 4b), with a mean deviation from the (101̅) plane of 0.096 Å. There are six hydrogen bonds in total, which link the two independent molecules: there are no such interactions between symmetry-equivalent molecules. The hydrogen bonds can be subdivided into four N–H**···**O interactions that link pyrimidinedione groups and two N–H**···**N contacts between imidazole rings. The hydrogen bonded layers are approximately parallel with the (101̅) planes of the unit cell (Figure 4a). The separation between the layers is ∼3.2 Å, consistent with the presence of weak van der Waals’ interactions. The hydrogen bonding in the xanthine structure bears a high resemblance to that observed in hypoxanthine crystals which also has two N–H**···**N contacts between imidazole rings.^10,30^ However, due to the lack of a second oxygen in hypoxanthine, there are only two N–H**···**O interactions that link pyrimidinedione groups. Despite this, the strong similarities in hydrogen bonding lead to similar intraplanar molecular positions (Figures 4b and 4c). A comparison between molecules within each plane using a root-mean-squared deviation (RMSD) gave 0.216 Å (Figure S3.3). However, the stacking of the planes in xanthine and hypoxanthine is different. The consequence of this means unit-cell parameters between xanthine and hypoxanthine are similar, except for higher symmetry between layers in xanthine, which results in its *b*-axis (the unique axis along which acts the 2_1_ screw) being roughly double that of hypoxanthine. Unit-cell parameters of hypoxanthine are [*a* = 7.102(2) Å, *b* = 9.759(2) Å, *c* = 10.387(2) Å, α = 58.85(2)°, β = 67.64(2)°, γ = 72.00(2)°, *V* = 564.026 Å^3^].^10^ There is also some more limited resemblance to that of the chemically similar compounds adenine and guanine, both of which also adopt a two-dimensional network of hydrogen bonds, with each molecule in these cases linked together by eight and four hydrogen bonds, respectively. The adoption of a two-dimensional network of hydrogen bonds brings about a structural similarity between these compounds and xanthine, which is consistent with the observation of solid solutions consisting of guanine, hypoxanthine, and xanthine in biogenic crystals.^31^ The different hydrogen-bonding arrangements in xanthine and hypoxanthine compared to guanine and adenine are illustrated in Figure S3.4. In all cases, the hydrogen-bonded layers are quasi-planar, where molecules display varying degrees of tilt from the mean plane (Figure S3.5). Intermolecular interactions in polymorph II of adenine allow it to form chains of hydrogen-bonded molecules: the “ribbon”-like nature of the resulting chains allows a greater degree of flexibility for molecules to tilt out of plane (Figure S3.5), resulting in greater deviation from the mean plane of the layer (of up to 13.4°). In comparison, the hydrogen-bonded xanthine network has much less flexibility due to interactions involving all available hydrogen-bond donors, resulting in a lower deviation from the mean plane of the layer of 6.7°. Interestingly, although xanthine and hypoxanthine have similar intralayer molecular arrangements, the angle of deviation from the mean plane is greater in xanthine than in hypoxanthine (2.9°) likely due to differences in the interplanar stacking, causing different interactions with molecules above and below each plane.

While the final *R*-factor of 10.90% is low for a kinematically refined 3D-ED structure, it would be regarded as high for SCXRD. This discrepancy may be due to several factors. First, the use of a kinematical model for structure refinement, wherein multiple scattering events of the electron within the sample are not accounted for and which results in “scrambled” intensities.^32^ However, the use of continuous rotation electron diffraction integrates through the Bragg condition which minimizes these effects.^33^ Second, some beam damage is inevitable for organic pharmaceutical samples.^34^ Despite this, xanthine appears to be sufficiently beam-resistant to allow some high-order reflections (close to the refinement cutoff resolution of 0.9 Å) to still be visible toward the end of the data acquisition; reflections at a resolution of 0.74 Å were visible at the beginning of the tilt series. Third, inelastic scattering leads to an undesired background signal,^34,35^ although it has been suggested that this can lead to a reduction in dynamical scattering effects.^36^ This combination of multiple scattering, beam damage, and inelastic scattering leads to a deviation from the simple kinematical model on which calculated intensities are based.

Powder X-ray diffraction (PXRD) was used to establish consistency between nanocrystals studied using 3D-ED and the bulk sample. In-situ PXRD characterization was performed at the Diamond Light Source Synchrotron, Beamline I11 (see Note S4 in the Supporting Information). PXRD data shows the effects of preferred orientation, with the strongest peak corresponding to the (202̅) stacked planes (Figure S4.1). A Rietveld rigid body refinement was performed, starting from the model obtained from 3D-ED data. However, the small crystal size and significant preferred orientation led to difficulties in obtaining an entirely satisfactory fit. In particular, the strongly asymmetric peak shapes (line broadening anisotropy) indicate the presence of planar disorder,^37^ likely layer stacking disorders.^38,39^ Crucially, peaks were present that could not be fully accounted for by our monoclinic xanthine structure (labeled in Figure S4.1), and which we struggled to model even using a Pawley fit with optimized parameters for anisotropic and asymmetric peak broadening (see the Supporting Information). This strongly suggests that other phases are present in the bulk xanthine powder, in addition to planar defects. Considering the purported chemical purity of the sample, our interpretation is that the analyzed powder contains other solid-state forms of xanthine, which is the subject of further work.^40^ This would also explain the nonideal Rietveld refinement of the PXRD data.

We postulate that, similar to the α- and β-phases of guanine,^11,41^ any such other (likely) polymorphs of xanthine consist of layers within which hydrogen bonding connectivity between xanthine molecules remain the same but that differ by the relative offset of adjacent layers. This higher-order structural variation of a polymorph is commonly known as polytypism, whereby differences in crystal structures are solely caused by the difference in the stacking sequence of layers. However, although the nature of hydrogen bonding interactions will likely remain the same, the deviation of each molecule from the mean plane of the layer will not necessarily be preserved, as is the case in guanine (Figure S3.5 c-d). Therefore, we propose to name our monoclinic polymorph of xanthine presented in this work the Form I polymorph.

## Concluding Remarks

This work presents the first reported crystal structure determination of xanthine, the Form I polymorph, which has been achieved using 3D-ED techniques. As might be expected, the structure shows structural similarities to that of guanine. Its highly planar nature also lends itself to potential low energy planar defects and further, the existence of other polymorphs, which is suggested by the PXRD data. In addition, the data were of sufficient quality to allow all hydrogens to be located and independently kinematically refined without the use of geometric restraints. This work builds upon the use of 3D-ED to determine the crystal structure of β-guanine^21^ and unlocks possibilities for the structural comparison between biogenic and synthetic xanthine crystals which may help understand mechanisms behind crystal morphogenesis and the morphological and functional diversity of biocrystals.^42^

Subsequent analysis using different acquisition modalities of electron diffraction such as scanning transmission electron microscopy techniques (4D-STEM)^43^ may also be able to investigate the planar disorder evident in PXRD data, while relating this to the crystal morphology and subcrystal structure of xanthine particles. In the future, aided by structural insights from 3D-ED, the combined use of multidimensional electron diffraction techniques could yield fresh insights into the microstructure of small organic molecules.

## Supporting Information Available

Information, tables, and figures to support the main text (PDF)
TOPAS input file from PXRD Rietveld Refinement (ZIP)

## Footnotes

1 , a measure of the precision of resulting mean intensities.^44^
