# Supplementary material for: Revealing the Crystal Structure of the Purine Base Xanthine with Three-Dimensional (3D) Electron Diffraction
Source: Cryst Growth Des. 2025 Feb 11;25(5):1293–8. doi: 10.1021/acs.cgd.4c01594 (PMC11887060; doi:10.1021/acs.cgd.4c01594)
Supplement: Supplementary file 1 — cg4c01594_si_001.pdf [file cg4c01594_si_001.pdf]

Supporting Information

Revealing the Crystal Structure of the Purine Base Xanthine with 3D Electron Diffraction

Helen W. Leung[a]*, Royston C. B. Copley[b], Giulio I. Lampronti[a], Sarah J. Day [c], Lucy K. Saunders[c], Duncan N. Johnstone[b], Paul A. Midgley[a] *

[a] Department of Materials Science and Metallurgy
University of Cambridge
27 Charles Babbage Road, Cambridge, CB3 0FS, United Kingdom

[b] GSK R&D
Gunnels Wood Road, Stevenage, SG1 2NY, United Kingdom

[c] Diamond Light Source Ltd, Beamline I11
Harwell, Oxford, United Kingdom

*corresponding authors: hl585@cam.ac.uk pam33@cam.ac.uk

S1. Sample Preparation of Xanthine Crystals for 3D-Electron Diffraction

Initially, xanthine powder (purchased from Sigma Aldrich, X7375, batch WXBD7599V) was directly deposited on Quantifoil grids. However, attempts at 3D-ED resulted in polycrystalline rings from sub-micron areas, suggesting that multiple crystals of different orientations were aggregated. Multiple sample preparation methods were attempted to grow single crystals or separate the crystals. Attempts to grow crystals from solution were unsuccessful and resulted in further polycrystallinity. An aqueous suspension of xanthine powder was made using 0.5 mg of xanthine in 14.5 ml of distilled water. 5 μL of this suspension was micro-pipetted and dropped directly onto Quantifoil grids. Grids were left in a fume cupboard to allow the water to evaporate under room temperature conditions. This left behind more evenly distributed xanthine crystals sufficiently isolated for 3D-ED.

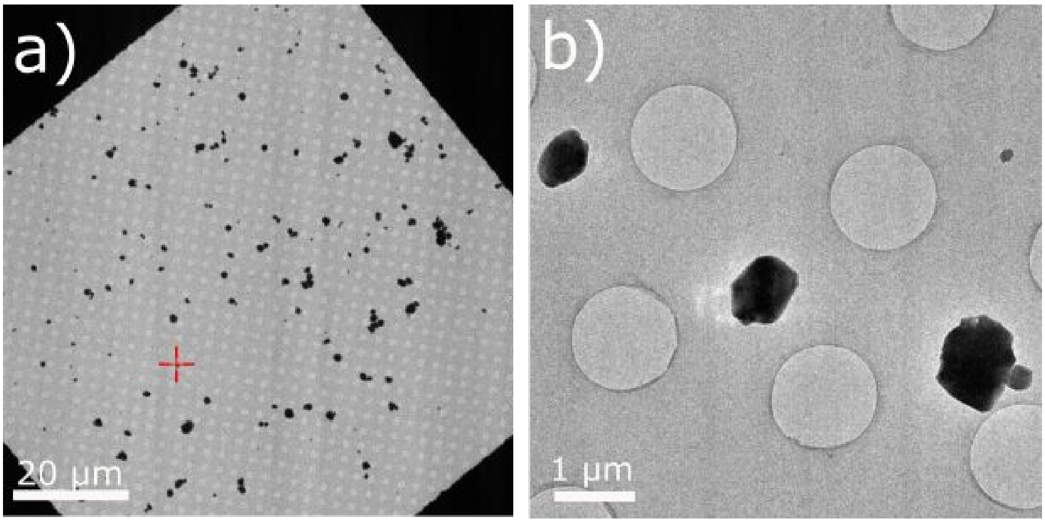

Figure S1.1: (a) Typical distribution of xanthine crystals on the grid resulting from directly depositing an aqueous suspension of xanthine powder onto grids. (b) Xanthine crystals displayed a block-like morphology.

S2. 3D-Electron Diffraction Data Collection and Data Processing

Continuous rotation 3D-ED was performed using a Thermo Fisher Titan Krios G3i electron microscope operated at 300 kV under cryogenic conditions. A diffraction pattern was recorded for each tilt increment over a range of ± 60 ° at a continuous tilt rate of 1 ° s^{-1}. Diffraction patterns were recorded on a CETA-16M camera with an exposure time of 0.5 s per frame, forming a tilt series of 240 diffraction patterns. These conditions result in a cumulative dose of 20 e $Å^{-2}$. The camera length was set such that Bragg spots corresponding to a resolution up to 0.7 Å could be detected. EPU-D software was used for the acquisition, making use of the auto-eucentric height function to minimise sample movement when tilting to high angles. 3D-ED data were collected from crystals with a cross-section of ca. 1 μm x 1 μm.

3D-ED data from particles which were single crystals were selected for further analysis. For these datasets, the tilt series was used to reconstruct reciprocal space, indexed, and integrated using Rigaku CrysAlisPro 1.171.43.110a software[1]. Systematic absences in the reciprocal

space sections were used to deduce likely space groups. These were consistent with the $P2_1/c$ space group (unique *b*-axis).

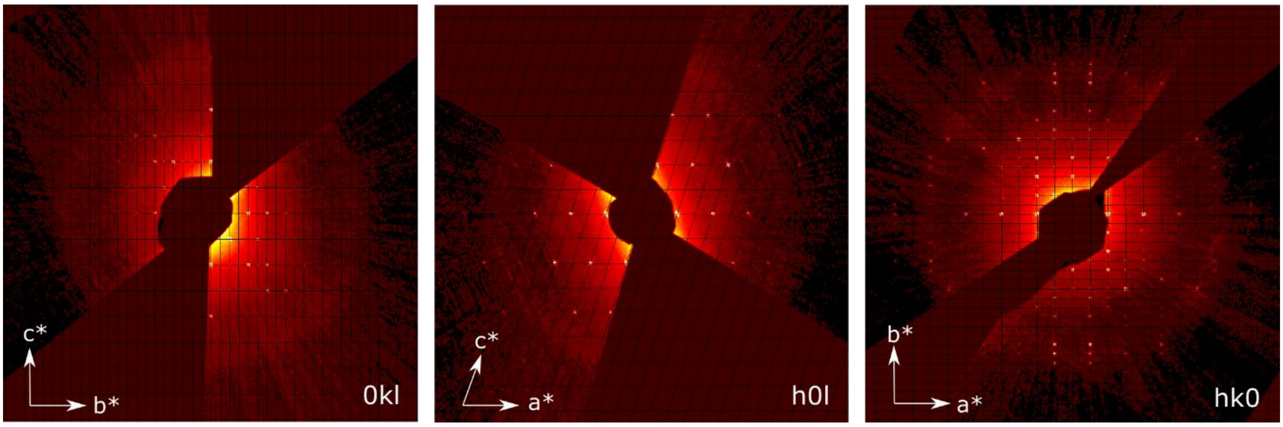

Figure S2.1: a) 0*kl*, b) *h*0*l*, c) *hk*0 slices from reconstructed reciprocal space as shown in Figure 2 in the main text. The reciprocal lattice grid is overlaid onto the patterns to show the size of reciprocal lattice vectors.

S3. Structure Solution and Refinement

The best dataset was used for structure solution and kinematical refinement with the $P2_1/c$ space group. SHELXD and SHELXL were accessed via the Olex2[2] graphical user interface. Structure visualisation and analysis was done using Mercury software[3].

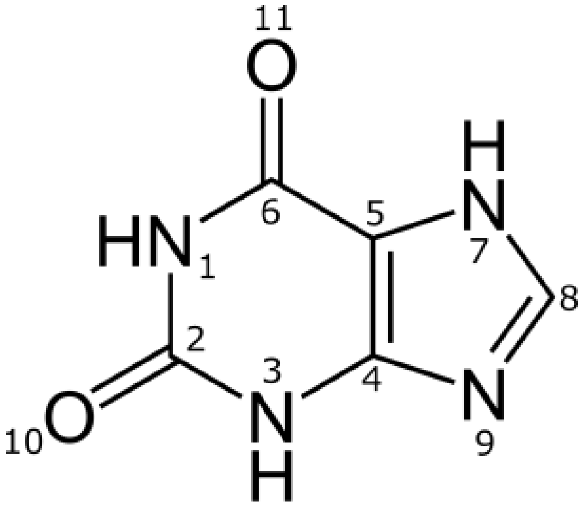

Figure S3.1: Labels used for structure refinement of xanthine. The 2nd xanthine molecule in the asymmetric unit has identical labels, except starting from N21 (20 is added to all numbers).

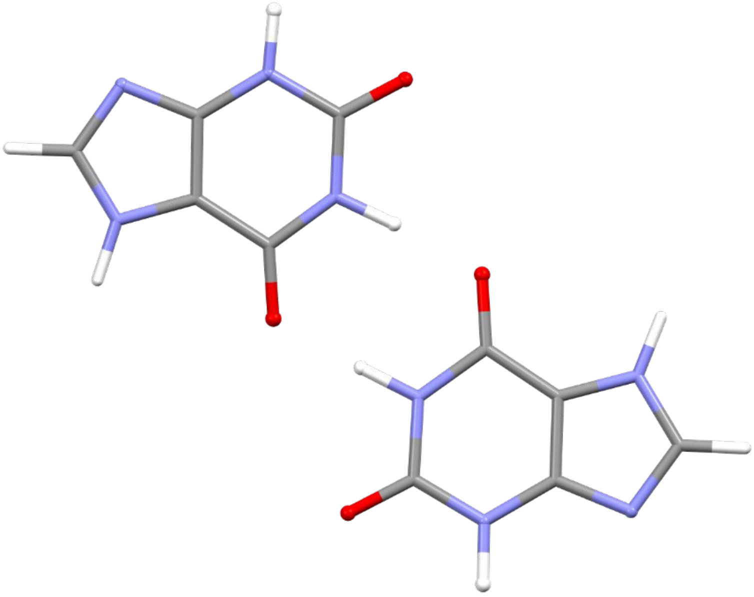

Figure S3.2: “Ellipsoids” show isotropic refinement of atomic displacement parameters (ADP) for all atoms in both xanthine molecules in each asymmetric unit, including hydrogens. A common isotropic ADP was refined for chemically equivalent non-hydrogen atoms e.g. N1 and N21, C2 and C22 etc. The hydrogen atoms on C8 and C28 were refined using idealised geometries, still allowing the C-H distances to refine but with a restraint to hold these to be equivalent. A similar restraint was applied to the chemically equivalent N-H distances e.g. N1-H1 and N21-H21 etc. Apart from this distance equivalence restraint, the heteroatom hydrogen coordinates were freely refined and not further geometrically idealised. For all hydrogen atoms, the isotropic ADP was a 1.2 multiple of that for the bonded atom. Atoms are coloured as follows: nitrogen (blue), oxygen (red), carbon (grey), and hydrogen (white). Isotropic atomic displacement factors were preferred because anisotropic refinement was not found to significantly improve the model.

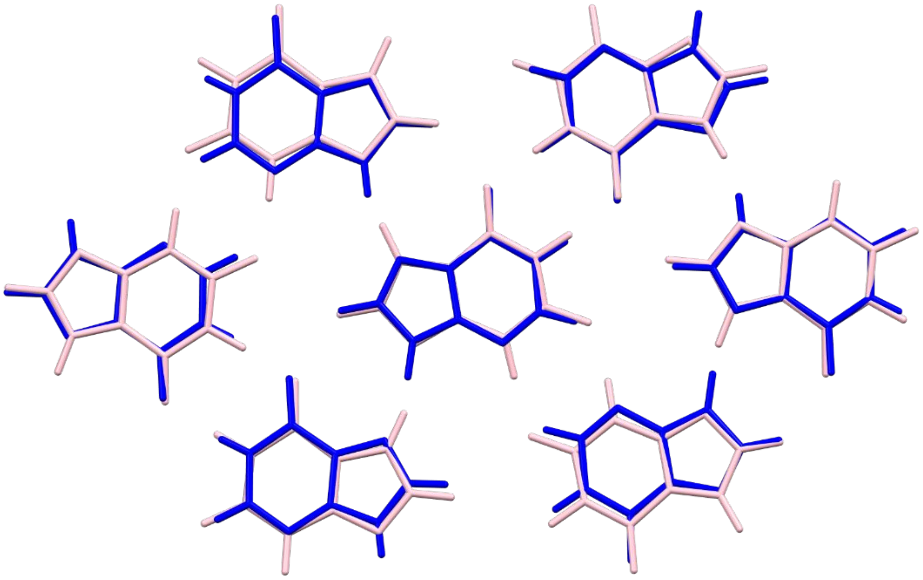

Figure S3.3: Visual overlay of planes of xanthine and hypoxanthine, showing the similarity of their in-plane packing. Xanthine molecules are shown in pink, whilst hypoxanthine molecules are shown in dark blue. The two molecules in this plane have

RMSD of 0.216 Å. In contrast, inter-planar positioning of these layers is not the same and there is no overlap. This analysis was done using the 'crystal packing similarity' function in Mercury[3] to compare the two structures.

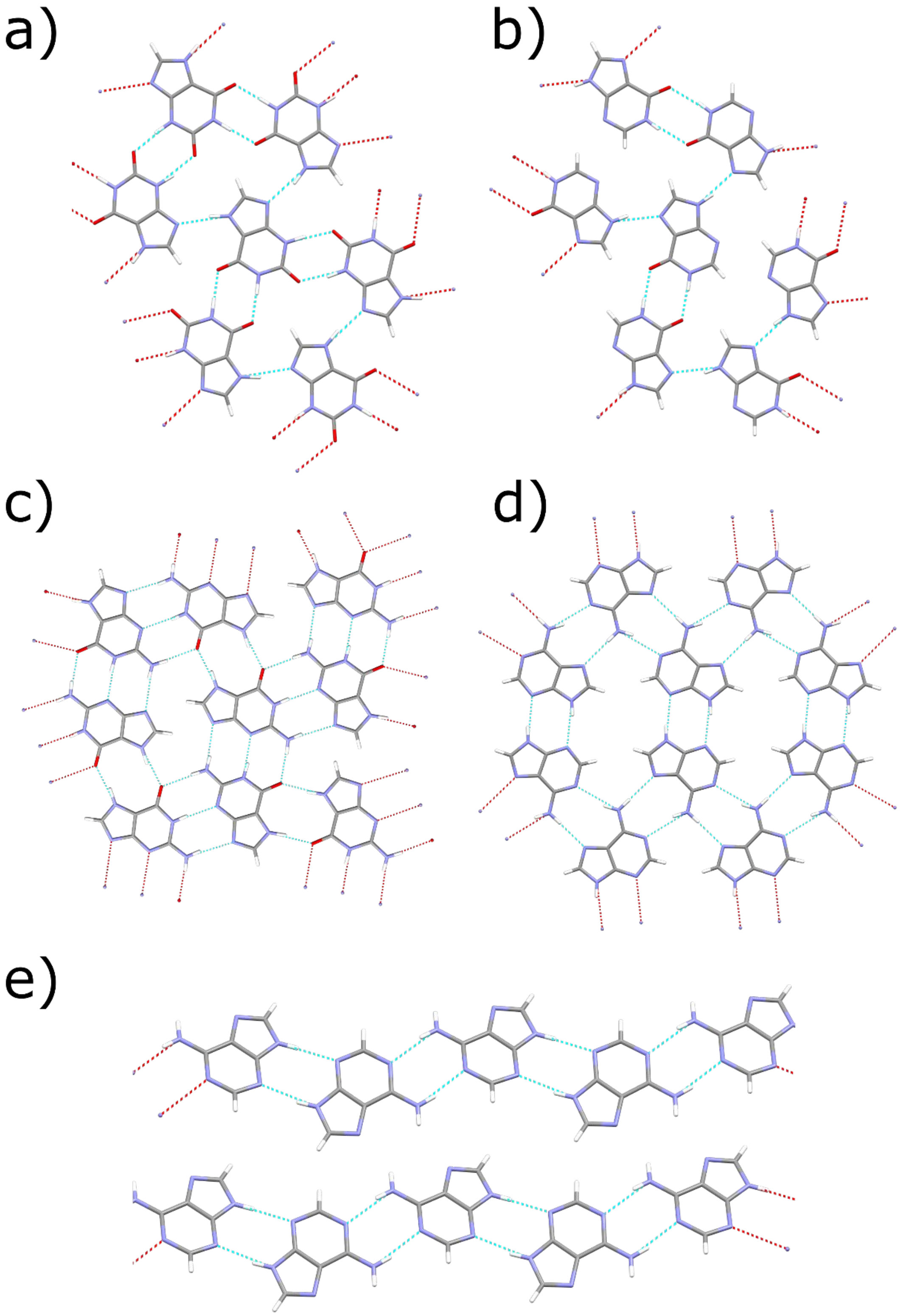

Figure S3.4: Hydrogen bonding within the group of planar structures (a) xanthine (b) hypoxanthine (c) α guanine (there are identical interactions in β guanine) (d) monoclinic adenine (e) orthorhombic adenine.

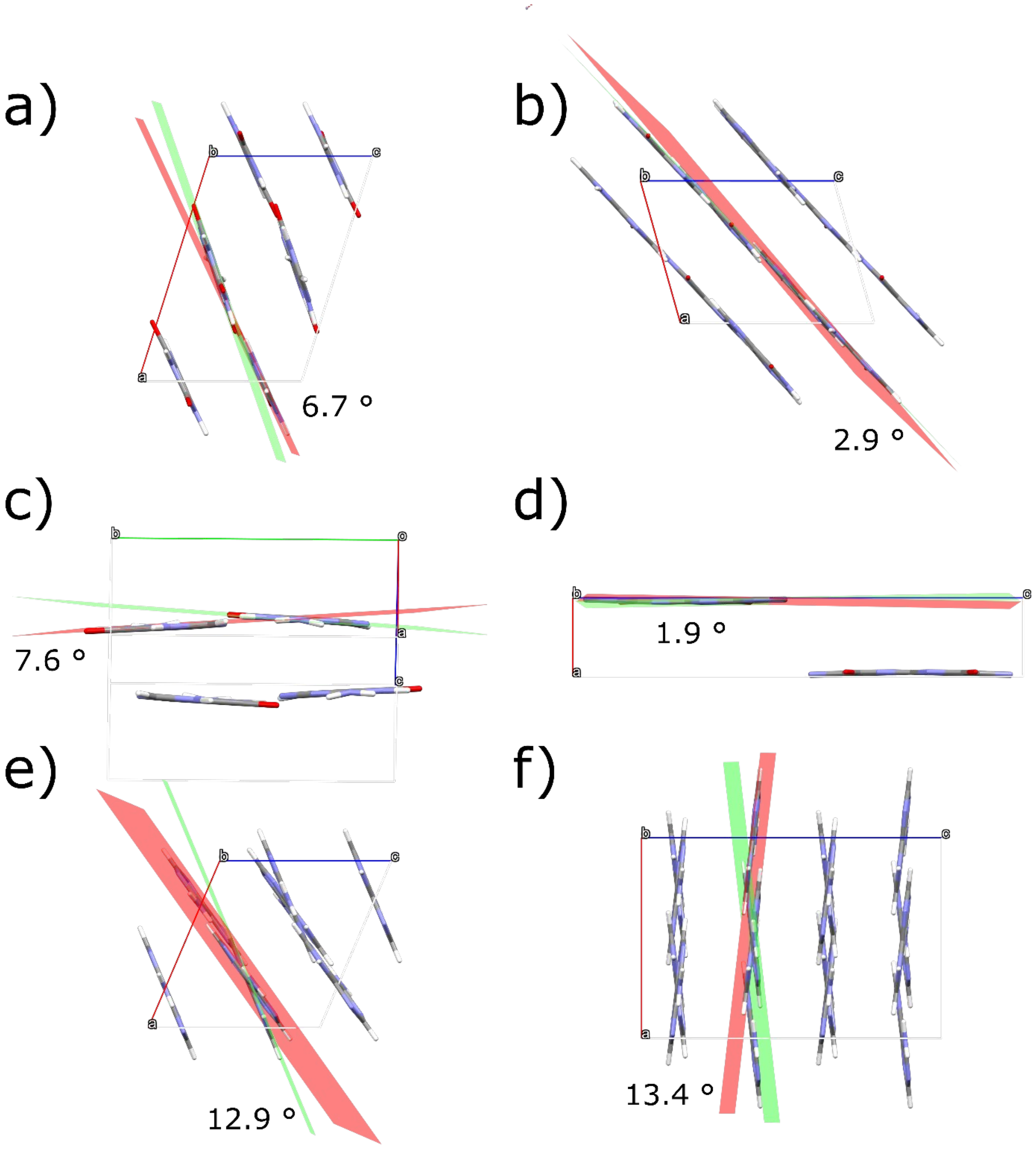

Figure S3.5: Side-on view of planar structures with mean planes drawn through molecules pictured in red and green. The angle between the red and green planes is shown for: (a) xanthine (b) hypoxanthine (c) α guanine (d) β guanine, (e) monoclinic adenine (f) orthorhombic adenine.

Identification code	xanthine_form_I	
Empirical formula	C5 H4 N4 O2	
Formula weight	152.11	
Temperature	87(2) K	
Wavelength	0.0197 Å	
Crystal system	Monoclinic	
Space group	P 21/c	
Unit cell dimensions	a = 9.82(11) Å	α= 90°.
	b = 17.87(8) Å	β= 107.5(9)°.
	c = 6.79(13) Å	γ = 90°.
Volume	1136(26) $Å^3$	
Z	8	
Density (calculated)	1.778 Mg/m^3	
Absorption coefficient	0.000 mm^{-1}	
F(000)*	217	
Crystal size	0.0004 x 0.0002 x 0.0001 mm^3	
Theta range for data collection	0.087 to 0.627°.	
Index ranges	-10<=h<=10, -19<=k<=19, -7<=l<=7	
Reflections collected	3851	
Independent reflections	1351 [R(int) = 0.1270]	
Completeness to theta = 0.627°	83.0 %	
Refinement method	Full-matrix least-squares on F^2	
Data / restraints / parameters	1351 / 4 / 99	
Goodness-of-fit on F^2	1.124	
Final R indices [I>2sigma(I)]	R1 = 0.1090, wR2 = 0.2489	
R indices (all data)	R1 = 0.1716, wR2 = 0.2941	
Extinction coefficient	914(44)	
Largest diff. peak and hole	0.148 and -0.195 e.$Å^{-3}$ ǂ	

Table S3.1: Selected parameters from the structure refinement using SHELXL, generated by XCIF. The structure solution and refinement executed here makes use of workflows which come from X-ray crystallography protocols. As such, we recognise that some parameters, such as the semi-empirical absorption corrections which likely account for effects of beam damage, are not ideal for use with electron diffraction. *This number represents the sum of the electron structure factors at a zero scattering angle. ǂ These units which result from SHELXL refer to X-ray scattering factors. However, electron scattering factors are used here, so the units that are correct should be $Å^{-2}$.

Bond	Bond length [Å]
N(1)-C(2)	1.37(2)
N(1)-C(6)	1.382(11)
N(1)-H(1)	1.08(2)
C(2)-O(10)	1.241(10)
C(2)-N(3)	1.372(12)
N(3)-C(4)	1.374(11)
N(3)-H(3)	1.05(3)
C(4)-N(9)	1.364(11)
C(4)-C(5)	1.38(2)
C(5)-N(7)	1.371(11)
C(5)-C(6)	1.438(13)
C(6)-O(11)	1.231(19)
N(7)-C(8)	1.352(17)
N(7)-H(7)	1.05(2)
C(8)-N(9)	1.348(14)
C(8)-H(8)	1.10(2)
N(21)-C(22)	1.37(2)
N(21)-C(26)	1.393(11)
N(21)-H(21)	1.08(2)
C(22)-O(30)	1.230(10)
C(22)-N(23)	1.368(12)
N(23)-C(24)	1.373(11)
N(23)-H(23)	1.05(3)
C(24)-N(29)	1.363(11)
C(24)-C(25)	1.39(2)
C(25)-N(27)	1.370(11)
C(25)-C(26)	1.436(13)
C(26)-O(31)	1.233(18)
N(27)-C(28)	1.352(17)
N(27)-H(27)	1.05(2)
C(28)-N(29)	1.359(14)
C(28)-H(28)	1.10(2)

Table S3.2. Bond lengths [Å] for xanthine Form I

S4. X-ray Powder Diffraction Studies

In-situ XRPD was performed at I11 Diamond synchrotron. Xanthine powder was loaded into a 0.5 mm borosilicate glass capillary and analysed using an X-ray beam of 0.82408 Å wavelength (15keV energy), refined using a NIST SRM640c Si standard. The sample was cooled to 80 K, consistent with 3D-ED measurement conditions, using a Cryostream Plus. Measurements were made using the Mythen wide-angle position sensitive detector (PSD).

With the monoclinic crystal structure of xanthine having been obtained using 3D-ED methods, we used XRPD to compare the bulk sample to the micro-crystals studied with 3D-ED. Rietveld refinement was carried out using TOPAS academic software[4], which refines to R_{wp} = 5.97% and GoF = 8.27%. The measurement of an empty capillary was used as an empirical background with a scale factor which was set as a refinable parameter.

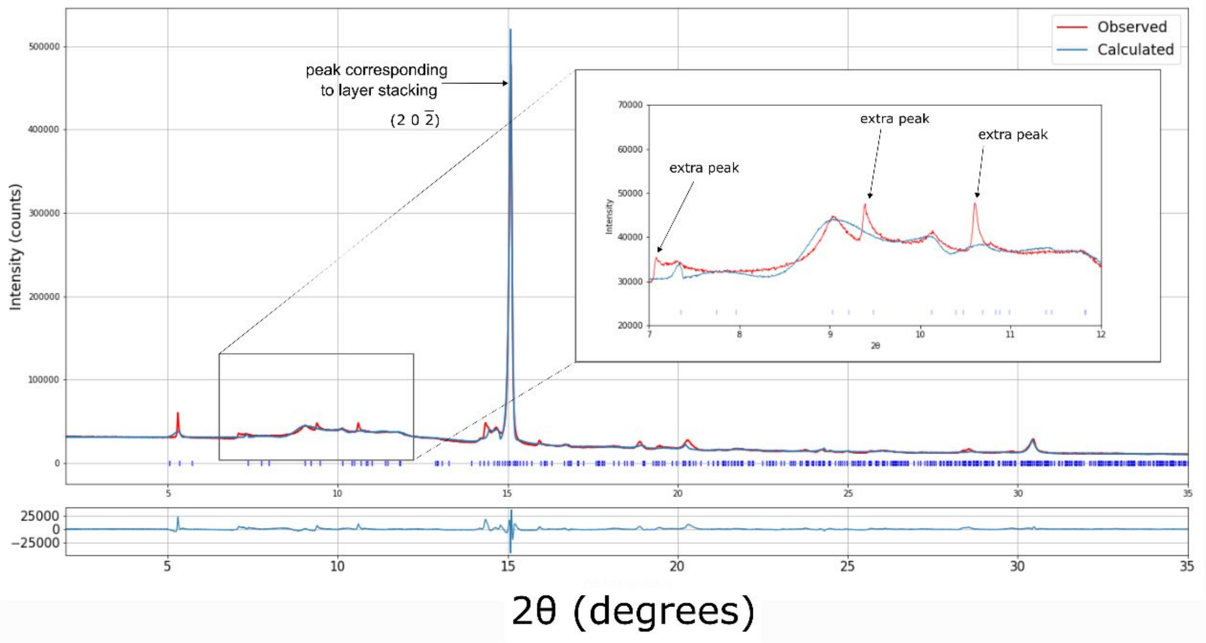

Figure S4.1: In-situ high resolution XRPD data. Whilst a general fit indicates the monoclinic 3D-ED structure is present in the bulk xanthine sample, the presence of unexpected peaks (see inset) and asymmetric peak shapes suggests the presence of multiple phases and planar disorder which are not accounted for by the single 3D-ED structure.

(1) Rigaku Oxford Diffraction. CrysAlisPro 1.171.43.110a. 2024.

(2) Dolomanov, O. V.; Bourhis, L. J.; Gildea, R. J.; Howard, J. A. K.; Puschmann, H. OLEX2: A Complete Structure Solution, Refinement and Analysis Program. *J. Appl. Crystallogr.* **2009**, *42* (2), 339–341. https://doi.org/10.1107/S0021889808042726.

(3) MacRae, C. F.; Sovago, I.; Cottrell, S. J.; Galek, P. T. A.; McCabe, P.; Pidcock, E.; Platings, M.; Shields, G. P.; Stevens, J. S.; Towler, M.; Wood, P. A. Mercury 4.0: From Visualization to Analysis, Design and Prediction. *J. Appl. Crystallogr.* **2020**, *53* (1), 226–235. https://doi.org/10.1107/S1600576719014092.

(4) A. Coelho. TOPAS-Academic V5 . 2012. https://www.bibsonomy.org/bibtex/882c0982cdc4ba90122dc5a61294f5af (accessed 2024-03-20).
